# Identifying Novel Drug Indications through Automated Reasoning

**DOI:** 10.1371/journal.pone.0040946

**Published:** 2012-07-23

**Authors:** Luis Tari, Nguyen Vo, Shanshan Liang, Jagruti Patel, Chitta Baral, James Cai

**Affiliations:** 1 Disease and Translational Informatics, Pharma Research and Early Development Informatics, Hoffmann-La Roche, Nutley, New Jersey, United States of America; 2 Department of Computer Science and Engineering, Arizona State University, Tempe, Arizona, United States of America; 3 Scientific Information Management, Pharma Research and Early Development Informatics, Hoffmann-La Roche, Nutley, New Jersey, United States of America; Wayne State University School of Medicine, United States of America

## Abstract

**Background:**

With the large amount of pharmacological and biological knowledge available in literature, finding novel drug indications for existing drugs using *in silico* approaches has become increasingly feasible. Typical literature-based approaches generate new hypotheses in the form of protein-protein interactions networks by means of linking concepts based on their cooccurrences within abstracts. However, this kind of approaches tends to generate too many hypotheses, and identifying new drug indications from large networks can be a time-consuming process.

**Methodology:**

In this work, we developed a method that acquires the necessary facts from literature and knowledge bases, and identifies new drug indications through automated reasoning. This is achieved by encoding the molecular effects caused by drug-target interactions and links to various diseases and drug mechanism as domain knowledge in AnsProlog, a declarative language that is useful for automated reasoning, including reasoning with incomplete information. Unlike other literature-based approaches, our approach is more fine-grained, especially in identifying indirect relationships for drug indications.

**Conclusion/Significance:**

To evaluate the capability of our approach in inferring novel drug indications, we applied our method to 943 drugs from DrugBank and asked if any of these drugs have potential anti-cancer activities based on information on their targets and molecular interaction types alone. A total of 507 drugs were found to have the potential to be used for cancer treatments. Among the potential anti-cancer drugs, 67 out of 81 drugs (a recall of 82.7%) are indeed known cancer drugs. In addition, 144 out of 289 drugs (a recall of 49.8%) are non-cancer drugs that are currently tested in clinical trials for cancer treatments. These results suggest that our method is able to infer drug indications (original or alternative) based on their molecular targets and interactions alone and has the potential to discover novel drug indications for existing drugs.

## Introduction

The current model of drug discovery and development is perceived as a costly and time-consuming process [1]. To reduce the cost and shorten the duration for drug development, drug repurposing, also known as drug repositioning, has become an attractive alternative to traditional drug development aiming to shorten the development process. Drug repurposing is the process of finding a new indication for existing drug compounds. In other words, it is a discovery process on how an existing drug compound can be used for the treatment of diseases other than its original indication. Reusing these drug compounds has the advantage of bypassing many of the expensive steps of drug development, such as in vitro and in vivo screening, chemical optimization, toxicology, bulk manufacturing, formulation development. This reduces cost and development risks, as well as shortens the typical 10–17 year process of drug development to 3–12 years [2]. The best known success story of drug repositioning is the development of sildenafil, a compound that was developed by Pfizer and intended for the treatment of angina. Clinical trials of the drug showed unexpected side effects that led to the treatment of erectile dysfunction, and sildenafil became the blockbuster drug more commonly known as Viagra®. Further studies and repositioning of the drug compound showed yet another therapeutic indication for treating pulmonary arterial hypertension, marketed as Revatio®. This is due to the fact that sildenafil is an inhibitor of phosphodiesterase-5 (PDE-5) proteins, and PDE-5 is known to be expressed in pulmonary hypertensive lungs [3].

The main concept behind drug repurposing is that novel drug indications can be identified based on the principle that the primary target of a drug can be associated with diseases other than its original drug indication. In addition, as drugs can act on multiple targets, secondary targets can be utilized for novel drug indications as well. Several systematic approaches of finding new uses for old drugs have been proposed. These methods can be broadly classified into two categories: target discovery based on chemical compound similarity [4] and literature-based discovery [5]. Compound similarity has been a popular approach to identify drug targets for drug repurposing. The assumption is that similar drug compounds have similar targets so that targets that are not shared between a pair of similar compounds can be identified as novel targets to the other. By identifying new targets for existing compounds, new drug indications can then be proposed. On the other hand, typical text mining methods focus on the extraction of knowledge such as protein-protein interactions from biomedical literature. These text mining efforts including the BioCreAtIvE challenge [6], a community effort that aims to advance the development of biological knowledge extraction systems, focus on the extraction of biological knowledge that is explicitly stated in the literature. Literature-based discovery methods go a step further by identifying relevant knowledge through text mining so that new knowledge can be inferred from existing knowledge. Swanson’s ABC Model [7] is a popular literature-based discovery methodology that was proposed to link two concepts through a commonly shared concept. Scientific concepts *A* and *C* form a relationship when concept *A* cooccurs with concept *B* in one publication while concepts *B* and *C* cooccur in another publication. Variations of Swanson’s ABC models have been described in the literature for the identification of indirect relationships [8], [9]. However, approaches based on cooccurrences of concepts within abstracts tend to generate too many hypotheses. Another direction for network-based approaches aims to uncover knowledge through the creation of biological networks. STITCH [10] and ChemProt [11] are examples of network-based approaches that take interactions extracted from literature and integrates with data from biological knowledge bases to create chemical compound-protein interaction networks. This kind of approach in linking the concepts does not consider the inherent relationships between the pairs of concepts such as interaction type and directionality of interactions, thus leading to a large number of hypotheses. To handle large networks that are generated by means of literature mining and other data sources, visualization tools have been proposed to assist the discovery of novel drug indications [12], [13].

In this paper, we propose a new literature-based discovery approach for drug repurposing that integrates facts from various sources to infer novel indications by means of automated reasoning. Our approach captures the various effects of drug-target interactions inside cells as well as the molecular mechanisms of diseases. Using cancer as an example, we utilized the wealth of knowledge about cancer and encoded oncogenes and tumor suppressors as well as cancer-related biological processes as the domain knowledge for our method. Together with the protein-protein interactions and gene-disease associations acquired from the literature, our approach identified drugs that are potential candidates for the treatment of cancer. By considering the interaction types and their directionality and the domain knowledge involved in the mechanism of action of drugs, our approach aims to produce biologically meaningful hypotheses for novel drug indications and can significantly reduce the number of hypotheses as compared to previous text mining and literature-based discovery approaches.

**Table 1:** Different types of knowledge used in our approach and their sources.

Types of knowledge	Sources
Drug-target interactions	DrugBank
Oncogenes and tumor suppressors	UniProt, Entrez Gene, CancerQuest
Genes involved in cancer-related biological processes	Gene Ontology
Gene-disease relations	Medline abstracts by text mining
Protein-protein interactions	Medline abstracts by text mining

## Methods

Our approach can be divided into three main components: (i) the *knowledge acquisition component*; (ii) the *knowledge representation component*; and (iii) the *reasoning component*. The knowledge acquisition component includes publicly available curated sources as well as the relevant facts for the identification of drug indications acquired using text mining. To automatically propose alternative drug indications, it is necessary to first represent the mechanism of drug action in the form of logic rules. With the facts acquired from the knowledge acquisition component and the logic rules defined in the knowledge representation component, the reasoning engine utilizes the logic rules to find interactions that link drugs with the corresponding drug indications.

**Table 2:** Examples of extracted gene-disease relationships and protein-protein interactions with their support evidences.

Evidences	Extracted relationships
The results of our study demonstrate that AMACR expression is *upregulated* ingastric cancer (PMID: 18787636)	<over-expressed AMACR, associated with, gastric cancer>
Therefore, *inactivation* of Rb protein by HPV 18 E7 protein may be associated withcarcinogenesis of small cell carcinoma (PMID:14506638)	<under-expressed RB1, associated with, small cell carcinoma>
Moreover, HER-2 expression was *stimulated* by EGF addition in young cells (PMID:8028398)	<EGF, induces, ERBB2>
*Inhibition* of PPARgamma activity by TNF-alpha is involved in pathogenesis of insulinresistance (PMID: 18655773)	<TNF, inhibits, PPARG>

### Mechanism of Action of Drugs

The basic mechanism of drug action involves the activation or inhibition of the function of drug targets that are responsible for certain diseases, and this interaction translates into clinical effects of the drug. One example is the drug levodopa, which is an agonist that targets the dopamine receptors to increase dopamine levels for the treatment of Parkinson’s disease [14]. Inhibition or activation of drug targets such as oncogenes and tumor suppressors can also trigger cancer-related biological processes and pathways. An example of such drug action is erlotinib, an antagonist that targets the oncogene known as the epidermal growth factor receptor (EGFR) and alters the signal transduction in the EGFR signaling pathway [15]. It is typical that a drug interacts with multiple targets, in which the original indication is related to the primary target. Alternative indications can be hypothesized through the secondary targets and their corresponding roles in diseases. On the other hand, a target can be involved in various diseases and biological pathways. By studying the roles of the target in diseases and pathways, alternative indications can be proposed through deeper understanding of the targets.

**Table 3:** Logic forms for the classes and entities involved in the drug mechanism domain.

Facts	Logic forms	Examples
*Prot* is a protein, e.g. P53	protein(Prot)	protein(tp53)
*Prot* is an oncogene, e.g. EGFR	oncogene(Prot)	oncogene(egfr)
*Prot* is a tumor suppressor, e.g. P53	suppressor(Prot)	suppressor(tp53)
*Dr* is a drug, e.g. moclobemide	drug(Dr)	drug(moclobemide)
*Dise* is a disease, e.g. depression	disease(Dise)	disease(depression)
*Bp* is a cancer-promoting biological process, e.g. positive regulation of cell proliferation	cancer_promoting_bioprocess(Bp)	cancer_promoting_bioprocess(pos_reg_cell_proliferation)
*Bp* is a cancer-resisting biological process, e.g. positive regulation of apoptosis	cancer_resisting_bioprocess(Bp)	cancer_resisting_bioprocess(pos_reg_apoptosis)

### Knowledge Acquisition

To identify novel drug indications, the first step of our approach is to acquire various types of knowledge that are relevant to the mechanism of action (MOA) of the drug. Such knowledge includes (i) drug-target interactions; (ii) oncogenes and tumor suppressors; (iii) genes involved in cancer-related biological processes; (iv) gene-disease relations; (iv) protein-protein interactions. Table 1 provides a summary of sources that are used to acquire knowledge for our approach. DrugBank [16] was used as the source of knowledge for drug-target relations, i.e. whether a drug is an antagonist or an agonist for a drug target. Several sources are utilized as there is no single source of complete knowledge on oncogenes and tumor suppressors. Specifically UniProt (http://www.uniprot.org), Entrez Gene (http://www.ncbi.nlm.nih.gov/gene) and CancerQuest (http://www.cancerquest.org/oncogene-table and http://www.cancerquest.org/tumor-suppressors-table) were considered in our approach. For UniProt and Entrez Gene, the list of cancer genes was obtained by using the keywords “oncogene” and “tumor suppressor” as search criteria. Genes belonging to cancer-related biological processes such as “cell proliferation”, “apoptosis” and “angiogenesis” were obtained from the Gene Ontology annotations (http://www.geneontology.org/GO.downloads.annotations.shtm).

While databases such as PharmGKB [17] and IntAct [18] are great resources for gene-disease relations and protein-protein interactions, such databases are limited in terms of the coverage of the literature due to the time-intensive process of manual curation. More importantly, it is commonly the case that the type of the interactions is not captured in these databases. This becomes an obstacle when the interactions from these databases are used in the discovery of new knowledge. Suppose we know that a protein interacts with an oncogene. The consequence of the interaction, i.e. whether the function of the oncogene is activated or suppressed due to the interaction, is an important factor when the interaction is considered as part of the mechanism of a drug for treating cancer. To capture the types of the interactions, our approach is to utilize text mining so that appropriate interactions can be identified efficiently from the literature.

Our text mining approach is to rely on grammatical structures and keywords to capture the directionality and the types of the interactions for the extraction of gene-disease relations and protein-protein interactions. The *parse tree query language (PTQL)* [19] is a suitable language that allows extraction patterns to be defined over keywords and grammatical structures. PTQL is a query language designed for information extraction over a database of syntactic structures of text known as the *parse tree database (PTDB)*. Our latest version of the PTDB contains a collection of 19 million Medline abstracts, and the Stanford parser [20] is utilized to create parse trees of sentences. BANNER [21] is used for the recognition of gene names from text, and the recognized gene names are then mapped to official gene symbols using GNAT [22]. By defining the keywords and extraction patterns in the form of PTQL queries, it becomes possible to extract the directionality and the types of interactions for gene-disease relations and protein-protein interactions. Specifically, the following types of interactions are extracted: (i) association of over- or under-expressed genes with diseases (denoted as <over−/under-expressed *p*, associated with, *d*>, where *p* corresponds to a gene/protein name and *d* for a disease name); (ii) stimulation or inhibition of proteins by other proteins (denoted as <*p1*, induces/inhibits, *p2*>, where *p1* and *p2* are gene/protein names and *p1* acts on *p2* in the interaction). Examples of these interactions are listed in Table 2.

### Knowledge Representation

To identify drug indications through automated reasoning, it is important to properly represent our knowledge on basic drug mechanism. This requires the formation of the *logic facts* for the knowledge acquired from various sources as described in the previous subsection. In addition, *logic rules* are used to represent the properties of drug mechanism. We adopted a popular knowledge representation language called AnsProlog [23], [24] for the representation of the logic facts and rules.

AnsProlog is a declarative language that is useful for reasoning, including reasoning with incomplete information. One of the advantages of using a declarative language is that we define what the program should achieve and not how it should be achieved. It is important to notice that AnsProlog is a declarative language different from Prolog. While Prolog is a programming language with roots in logic, it includes many non-logical features that are not declarative, making it unsuitable for knowledge representation. Here we give a brief introduction to the syntax of AnsProlog.

An *AnsProlog rule* is of the form:
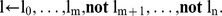
where *l_i_*s are literals and **not** represents *negation as failures*. The intuitive meaning of the above rule is that if it is known that literals *l*_0_,..., *l_m_* are to be true and if *l_m_*_+1_,..., *l_n_* can assume to be false, then *l* must be true. A literal is defined as either an atom or an atom preceded by the symbol ¬ that indicates *classical negation*. If there is no literal *l* in the head of a rule, then the rule is referred as a *constraint*. On the other hand, if there are no literals in the body of a rule, then the rule is referred as a *fact*, and its short hand of the representation of a fact is simply the head literal itself. An answer set program is composed of a set of AnsProlog rules, and the interpretation of an answer set program is called answer sets. Readers can refer to [25] for more details on the syntax and semantics of AnsProlog.

### Logic Facts

Two basic types of logic facts are represented in our drug mechanism domain: (i) entities and classes such as proteins and drugs that are involved in drug mechanism; (ii) interactions such as gene-disease relationships. The class protein is represented in the form of *protein(Prot)*, in which *Prot* is a variable for the class, and *protein(tp53)* is an instance of the class protein. The entities and their logic forms are shown in Table 3.

The class cancer-resisting biological process involves the following instances of Gene Ontology terms:

- Negative regulation of cell proliferation (GO:0008285)
- Positive regulation of apoptosis (GO:0043065)
- Negative regulation of angiogenesis (GO:0016525)

On the other hand, the class cancer-promoting biological process involves these instances:

- Positive regulation of cell proliferation (GO:0008284)
- Negative regulation of apoptosis (GO:0043066)
- Positive regulation of angiogenesis (GO:0045766)

For the interactions involved in the domain, they are represented with the predicate *interaction* for drug-target and protein-protein interactions and *relation* for gene-disease as well as gene-biological process relations. For instance, the logic form of the gene-disease relation <over-expressed AMACR, associated with, gastric cancer> is represented as relation(overexpressed(amacr), associated_with, gastric_cancer) while interaction(egf, induces, erbb2) is the logic form for the protein-protein interaction <EGF, induces, ERBB2>, and EGF is the *interactor* of the interaction that acts upon ERBB2, the *interactee* of the interaction. A complete list of logic forms for the interactions is shown in Table 4.

### Logic Rules

In representing the process of drug mechanism, logic rules are used to describe how a drug triggers the effect of the proteins based on the acquired interactions. Through the effects of the proteins, a series of steps eventually leads to the therapeutic relationship between the drug and the corresponding disease. We represent each triggering step on how a drug *Dr* affects the state of a protein *Prot* in the form of *trigger(Dr, Action, Prot, Step)*. *Action* is a class of effects such as *activates* (a drug activating a protein) and *inactivates* (a drug inhibiting a protein). *Step* is a variable indicating the order of the triggering step in the series. For instance, *trigger(moclobemide, inactivates, maoa, 1)* indicates that moclobemide inhibits the function of MAOA in step 1.

The core idea of the representation of mechanism of actions is to encode the pre- and post-conditions of interactions, also known as the executability and direct effects of actions. Using the effect of the activation of a tumor suppressor (denoted as *Prot*) as an example, cancer is identified as an indication for drug *Dr* when activation of the tumor suppressor is triggered by *Dr* previously. This mechanism is captured by the following AnsProlog rule:
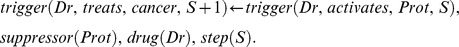



The principles behind the representation of mechanism of drug action are described below:

- Drug *Dr* triggers the inhibition (*respectively* activation) of protein *Prot* when *Dr* acts as an antagonist (*respectively* an agonist) for Prot. This is the initial step to trigger the mechanism.
- Drug *Dr* triggers the activation (*respectively* inactivation) of the function of protein *Prot2* when protein *Prot1* has been activated by *Dr* and the activated *Prot1* increases (*respectively* decreases) the expression of *Prot2.*
- Drug *Dr* is identified as a treatment for cancer when protein *Prot* has been inhibited (*respectively* induced) by *Dr* and overexpressed (*respectively* underexpressed) *Prot* is known to be associated with cancer.
- Drug *Dr* is identified as a treatment for cancer when oncogene *Prot* has been inhibited by *Dr*.
- Drug *Dr* is identified as a treatment for cancer when tumor suppressor *Prot* has been activated by *Dr*.
- Drug *Dr* is identified as a treatment for cancer when protein *Prot*, which is involved in cancer-promoting biological process, has been inhibited by *Dr*.
- Drug *Dr* is identified as a treatment for cancer when protein *Prot*, which is involved in cancer-resisting biological process, has been activated by *Dr*.

A list of AnsProlog logic rules describing the actions and effects involved in drug mechanism can be found in Tables S1 and S2 of the supplementary information.

**Table 4:** Logic forms for the interactions involved in the drug mechanism domain.

Relations	Logic forms
Drug *Dr* induces the activity of protein *Prot*	interaction(Dr, induces, Prot)
Drug *Dr* inhibits the activity of protein *Prot*	interaction(Dr, inhibits, Prot)
Protein *Prot1* induces the activity of Protein *Prot2*	interaction(Prot1, induces, Prot2)
Protein *Prot1* inhibits the activity of Protein *Prot2*	interaction(Prot1, inhibits, Prot2)
Overexpressed protein *Prot* is associated with disease *Dise*	relation(overexpressed(Prot), associated_with, Dise)
Underexpressed protein *Prot* is associated with disease *Dise*	relation(underexpressed(Prot), associated_with, Dise)
Protein *Prot* plays a role in biological process *Bp*	relation(Prot, is_associated, Bp)

**Figure 1:**
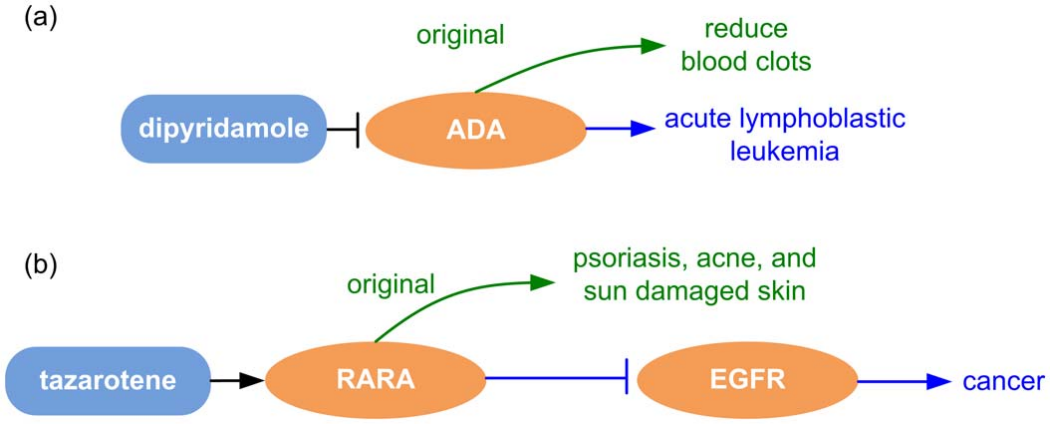
A diagrammatic view of (a) direct and (b) indirect inferences for dipyridamole and tazarotene as novel cancer indications.

**Table 5:** Evaluation of the inferences using a list of 943 drugs based on original indication and clinical trials.

	Cancer genes	GO	Text mining	All
Cancer as original indication (81)	25	43	58	67 (82.7%)
Non-cancer drugs under clinical trials for cancer (289)	46	95	133	144 (49.8%)
Total inferences	171	335	455	507
% inferences confirmed to be cancer-related	41.5%	41.2%	42.0%	41.6%

### Reasoning

With the acquired facts in logic form and the drug mechanism of actions described in logic rules, the next step is to define our goal – find a series of steps that eventually identifies a possible drug indication. Unlike semantic technologies such as SPARQL where the user has to explicitly define the right kind of queries in order to link up various sources of knowledge, the AnsProlog logic rules defined in the previous section only describe the effects of actions for the next step given the state of the current step and the logic facts. It is the task of the reasoning component to link up various sources and assign ordering of the steps that lead to a series of steps for drug indication. Our expectation is that the inference has to include: (i) a series of steps that involves a triggering step on how a drug *Dr* can be used for the treatment of cancer in the form of *trigger(Dr, treats, cancer, S)*; (ii) the triggering step *trigger(Dr, treats, cancer, S)* as the last step of the inference. To compute the answer sets that infer drug indications, an AnsProlog solver called clingo [26] is utilized to compute direct and indirect inferences based on the logic rules and the acquired logic facts.

**Figure 2:**
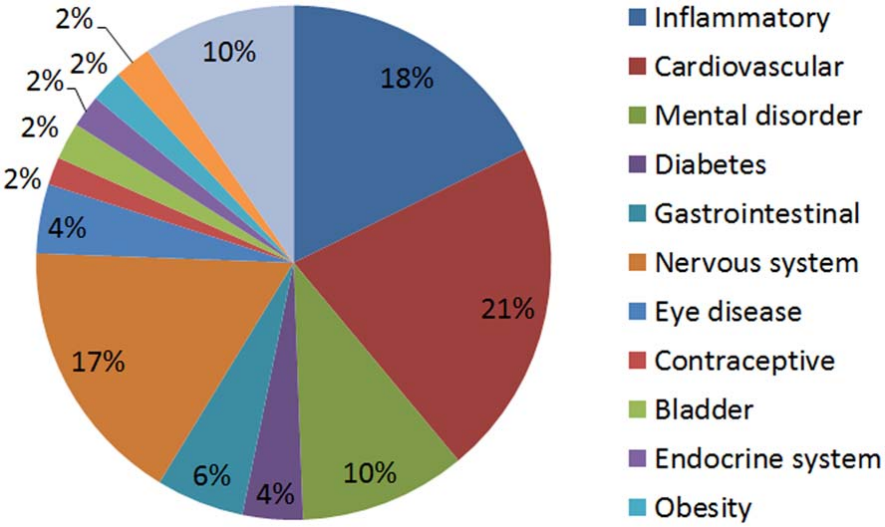
Treatment distribution for the 296 inferred drugs that neither have cancer as the original indication nor in clinical trials for cancer.

### Scenarios

Two types of inferences can be generated by our method: direct inference and indirect inference. Direct inference corresponds to drug indications that are directly triggered by drug targets, while drug targets play an indirect role in diseases in indirect inference. We illustrate each of the steps involved in inferring alternative drug indications for dipyridamole and tazarotene.

### Example of a Direct Inference: Dipyridamole as a Treatment for Leukemia

Dipyridamole is used to reduce blood clots through the inhibition of adenosine deaminase [PubMed-Health: Dipyridamole]. To find alternative indications for dipyridamole, the first step of our method is to acquire the necessary knowledge such as drug-target interactions and gene-disease relations. In this case, the following facts were acquired:

- interaction(dipyridamole, inhibits, ada): dipyridamole acts as an antagonist for adenosine deaminase (ADA) [Source: DrugBank]
- relation(overexpressed(ada), associated_with, cancer): *High levels* of adenosine deaminase (ADA) activity have been *associated with* normal T cell differentiation and T cell disease, such as acute lymphoblastic leukemia [Source: PMID: 6981287]

Dipyridamole is proposed as a potential treatment for cancer as ADA can be inhibited by dipyridamole and overexpression of ADA is associated with acute lymphoblastic leukemia. As of September 2011, dipyridamole is under phase I clinical trial for treatment of hepatic metastases from solid tumors [27].

- trigger(dipyridamole, inactivates, ada, 1)
- trigger(dipyridamole, treats, cancer, 2)

Figure 1 illustrates the steps involved in the direct inference of drug indication for dipyridamole.

### Example of an Indirect Inference: Tazarotene as a Treatment for Cancer

Tazarotene is approved for the treatment of psoriasis and acne. The facts below are acquired from different sources to identify alternative indication of tazarotene.

- interaction(tazarotene, induces, rara): tazarotene acts as an agonist for retinoic acid receptor alpha (RARA) [Source: DrugBank]
- interaction(rara, inhibits, egfr): These results suggest that RAR ligand-associated down-regulation of EGFR activity reduces cell proliferation by reducing the magnitude and duration of EGF-dependent ERK1/2 activation. [Source: PMID: 11788593]
- oncogene(efgr) [Source: CancerQuest]

With the acquired facts and the logic rules, the following steps in the inference are triggered:

- trigger(tazarotene, activates, RARA, 1)
- trigger(tazarotene, inactivates, EGFR, 2)
- trigger(tazarotene, treats, cancer, 3)

The indirect inference generated by our method shows that RARA can be activated by the agonist tazarotene. The activated RARA inhibits EGFR expression, and the inhibition of the oncogene EGFR can lead to cancer treatment. This inference is illustrated in Figure 1(b). As of April 2009, a phase II trial is currently underway to study the effectiveness of tazarotene in treating patients with basal cell skin cancer. The study is estimated to be completed by June 2013 [28].

## Results

For the knowledge acquisition component, we first compiled a list of drugs from DrugBank that contain information on their targets and interaction types, i.e. whether a drug is an antagonist or agonist for a target. This results in a list of 943 drugs that constitute 1704 drug-target interactions. In addition, a list of 265 cancer-related genes was obtained from UniProt, Entrez Gene and CancerQuest and another 1420 genes that are involved in cancer-related biological processes were acquired from the Gene Ontology. Together with 16816 protein-protein interactions and 25866 gene-disease relations extracted from the literature, these form a knowledge base of facts that are relevant to the mechanism of actions of drugs.

**Table 6:** Performance of the extraction of gene-disease relations (GDRs) and protein-protein interactions (PPIs).

	GDRs(Bundschus corpus)	PPIs (Bioinfer corpus)
True Positives (TP)	205	20
False Positives (FP)	14	18
False Negatives (FN)	469	150
Precision	93.61%	52.63%
Recall	30.42%	11.76%
F-measure	45.91%	19.23%

**Table 7:** Examples of incorrectly extracted gene-disease relations due to negation (E1) and wrong interactor (E2).

	Gene-disease relation	Sentence
E1	<overexpressed CCR7, associated with, lymphocyte-predominant Hodgkin disease>	*Up-regulation* of *CCR7* in classical but not in *lymphocyte-predominant Hodgkin disease* correlates with ….
E2	<overexpressed Bcl-2, associated with, acute myelogenous leukemia>	Synergistic *induction* of apoptosis by simultaneous disruption of the *Bcl-2* and MEK/MAPK pathways in *acute myelogenous leukemia*.

To assess the performance of our approach, our evaluation involves two aspects: (i) whether the drug indications suggested by our MOA-based approach are indeed the original indications of the drugs, without the direct use of such information; (ii) whether our suggested drug indications are currently under clinical trials for the indications according to ClinicalTrials.gov. Among the 943 drugs that were obtained from DrugBank, 81 of them are indicated as cancer drugs according to DrugBank. We also downloaded the records of the clinical trials from http://clinicaltrials.gov dated in December 2011. 289 drugs that do not have cancer as their original indications are found to be currently investigated as therapeutics for various types of cancers.

Our method suggested 507 drugs that have the potential to be used for cancer treatments. Among the suggested drug uses, 67 out of 81 drugs (a recall of 82.7%) are indeed drugs with cancer as their original indications. In addition, 144 out of 289 drugs (a recall of 49.8%) are non-cancer drugs that are in clinical trials for cancer. In other words, 211 out of the 507 inferred drug indications are confirmed to be cancer-related. These results, summarized in Table 5, show that our method is capable of assigning correct drug indications. We also compared the contribution in inferring drug indications among the various different sources of knowledge, i.e. the use of cancer-related genes (denoted as *Cancer genes*, genes involved in cancer-related biological processes (*GO*) and relations extracted from literature (*Text mining*). We found that the inferences generated based on each of the three sources has about the same reliability in terms of the number of inferences that are confirmed to be cancer-related. All three of them are in the range of 41% to 42%, as illustrated in Table 5. This shows that relations extracted by means of text mining can be as reliable as other sources for inference of alternative indications. With the broad coverage of relations obtained from text mining, findings for alternative indications can be more comprehensive than solely using manual curated sources.

We first performed analysis on the known cancer drugs that have been missed in our prediction. As indicated in Table 5, 67 of the 81 known cancer drugs are correctly predicted to be drugs for cancer treatment by our system. Among the 14 missed cancer drugs, interactions related to the drug targets of 8 of these cancer drugs cannot be found in the knowledge sources that were used by our system. The other 6 include contradictory interactions for the drugs and their drug targets within our knowledge sources. For example, PNP is one of the drug targets for cladribine, and it is known to be an agonist for PNP according to DrugBank. However, PNP is involved in the positive regulation of cell proliferation (GO:0042102) based on Gene Ontology. Activated PNP would lead to increase rate of cell proliferation, which is not ideal to be used for cancer treatment according to our system. Details of the analysis can be found in Table S3 of the supplementary information.

We further characterize the remaining 296 drugs that do not have cancer as original indications nor found to be in clinical trials for cancer. We first categorized the drugs in major treatment categories, and found that 17.7% of these drugs are currently used for treatments of inflammation. Links between Inflammation and tumor progression has been previously established in literature [29]. Another major category is the treatment of cardiovascular diseases constituting about 21.2%. The distribution of the main disease types is summarized in Figure 2.

### Evaluation of Text Mining Results

The inference of new indications for drugs largely depends on the correctness of the interactions extracted from the literature. Here we performed evaluation for the extraction of gene-disease relations and protein-protein interactions using various corpora. We adopted a corpus of gene-disease relations annotated from 5720 GeneRIF sentences [30] using the *altered expression* category for the evaluation of our gene-disease relations. The altered expression category contains 1044 gene-disease relations that correspond to the change of gene expression and its relations with diseases. In our evaluation we focused on relations that indicate overexpression or underexpression of genes to reflect our model of drug mechanism, and this forms a subset of 674 gene-disease relations. Our evaluation indicates that the extracted gene-disease relations result in a precision of 93.61%. The results of the evaluation are summarized in Table 6. Further analysis revealed that 50% of the incorrect gene-disease relations (i.e. false positives) are due to negation and another 28% of the false positives involved incorrect interactors or interactees in the extracted relations. Examples of incorrectly extracted gene-disease relations are shown in Table 7.

For protein-protein interactions, we performed the evaluation using the BioInfer corpus [31], one of the commonly used corpora for the evaluation of protein-protein interaction extraction. The BioInfer corpus contains 1100 sentences from Medline abstracts annotated with various biological relationships that include 425 protein-protein interactions. In this evaluation we focused on interactions that indicate the increase or decrease of the expression of a protein by another protein, and this forms a subset of 170 protein-protein interactions. Our evaluation indicates that the extracted protein-protein interactions results in a precision of 52.63%. The results of the evaluation are summarized in Table 6. Further analysis revealed that 50% of the false positives are due to incorrect interactee and another 27.8% of the false positives involved incorrect interactors. The rest of the false positives include both incorrect interactors and interactees such that the pair of entities has no actual relation to each other.

## Discussion

Automated reasoning is a powerful technique in artificial intelligence that enables knowledge inference based on domain knowledge and multiple data sources. In the biomedical domain the capabilities of reasoning have been demonstrated in the synthesis of pharmacokinetic pathways [32] and identification of drug-drug interactions [33]. Here we demonstrate the capability of automated reasoning to another important aspect of the drug development process – identification of novel drug indications for existing drugs. Unlike typical literature-based approaches that produce large network of biological entities based on coocurrences, our approach takes interaction types and directionality into consideration so that the search space is more computationally feasible. In addition, the hypotheses generated by our approach reflect the mechanism of action of drugs as well as the key mechanisms of cancer. This eliminates the time-consuming process of using network visualization to sift through the large network of interactions manually to identify novel drug indications. Our results showed that a significant number of drugs predicted by our method indeed have cancer as the original indication. Some of our findings even showed that the drugs are indeed currently under clinical trials for cancer.

While our method is capable of making not only correct but also novel drug indications, our current approach is limited to the identification of cancer treatment. In addition, the false positives for the relations obtained from text mining may contribute to the overall false positives in our predictions. Further improvement of our text mining method is needed to produce even more reliable inferences. To predict alternative indications for other disease areas, the domain knowledge has to be extended to encode the mechanism of other kinds of diseases and signaling pathways. Another future direction is to capture chemical structure information of drug compounds in order to identify alternative drug indications.

## Supporting Information

Table S1 A list of AnsProlog logic rules describing the actions and effects involved in mechanism of actions of drugs.
(DOC)

Table S2 Constraints that were used to define inference goals.
(DOCX)

Table S3 A list of 14 cancer drugs that were not identified by our approach.
(DOCX)
