# Supplementary material for: Identifying Novel Drug Indications through Automated Reasoning
Source: PLoS One. 2012 Jul 23;7(7):e40946. doi: 10.1371/journal.pone.0040946 (PMC3402456; doi:10.1371/journal.pone.0040946)
Supplement: Table S1 — A list of AnsProlog logic rules describing the actions and effects involved in mechanism of actions of drugs. (DOC) [file pone.0040946.s001.doc]

| **AnsProlog rule and its description** |
| --- |
| Rules 1a/1b: function of protein *Prot* is activated/inactivated at step 1 when drug *Dr* acts as an agonist/antagonist for *Prot*  trigger(Dr, activates/inactivates, Prot, 1) ← interaction(Dr, induces/inhibits, Prot), protein(Prot), drug(Dr). |
| Rules 2a/2b: drug *Dr* triggers the activation/inactivation of the function of protein *Prot2* in step *S*+1 when protein *Prot1* has been activated in the previous step *S* and the activated *Prot1* increases/decreases the expression of *Prot2*  trigger(Dr, activates/inactivates, Prot2, S+1) ← trigger(Dr, activates, Prot1, S), interaction(Prot1, induces/inhibits, Prot2), drug(Dr), protein(Prot1;Prot2), step(S). |
| Rules 3a/3b: Cancer is identified as an indication for drug *Dr* in step *S*+1 when protein *Prot* has been inhibited/induced in the previous step *S* and overexpressed/underexpressed *Prot* is known to be associated with cancer.  trigger(Dr, treats, cancer, S+1) ← trigger(Dr, inactivates/activates, Prot, S), relation(overexpressed/underxpressed(Prot),  associated_with, cancer), drug(Dr), protein(Prot), step(S). |
| Rule 4a: Cancer is identified as an indication for drug *Dr* in step *S*+1 when oncogene *Prot* has been inhibited in the previous step *S*.  trigger(Dr, treats, cancer, S+1) ← trigger(Dr, inactivates, Prot, S), oncogene(Prot), drug(Dr), step(S). |
| Rule 4b: Cancer is identified as an indication for drug *Dr* in step *S*+1 when tumor suppressor *Prot* has been stimulated in the previous step *S*.  trigger(Dr, treats, cancer, S+1) ← trigger(Dr, activates, Prot, S), suppressor(Prot), drug(Dr), step(S). |
| Rule 5a: Cancer is identified as an indication for drug *Dr* in step *S*+1 when protein *Prot*, which is involved in cancer-promoting biological process *Bp*, has been inhibited in the previous step *S*.  trigger(Dr, treats, cancer, S+1) ← relation(Prot, is_associated, Bp),  trigger(Dr, inactivates, Prot, S), protein(Prot), drug(Dr), cancer_promoting_bioprocess(Bp),step(S). |
| Rule 5b: Cancer is identified as an indication for drug *Dr* in step *S*+1 when protein *Prot*, which is involved in cancer-resisting biological process *Bp*, has been activated in the previous step *S*.  trigger(Dr, treats, cancer, S+1) ← relation(Prot, is_associated, Bp),  trigger(Dr, activates, Prot, S), protein(Prot), drug(Dr), cancer_resisting_bioprocess(Bp), step(S). |
