# Supplementary material for: Identifying Novel Drug Indications through Automated Reasoning
Source: PLoS One. 2012 Jul 23;7(7):e40946. doi: 10.1371/journal.pone.0040946 (PMC3402456; doi:10.1371/journal.pone.0040946)
Supplement: Table S2 — Constraints that were used to define inference goals. (DOCX) [file pone.0040946.s002.docx]

| **AnsProlog rule and its description** |
| --- |
| Rule 6: an inference is valid only if goal becomes true, i.e. the series of steps includes the triggering step *trigger(Dr, treats, cancer, S)*.  goal ← trigger(Dr, treats, cancer, S), drug(Dr), step(S).  ← not goal. |
| Rule 7: no other interactions should follow *trigger(Dr, treats, cancer, S)* in a valid inference.  ← trigger(Dr, activates/inactivates, Prot, S), trigger(Dr, treats, cancer, S1), S >= S1, protein(Prot), drug(Dr), step(S;S1). |
