# Supplementary material for: Identifying Novel Drug Indications through Automated Reasoning
Source: PLoS One. 2012 Jul 23;7(7):e40946. doi: 10.1371/journal.pone.0040946 (PMC3402456; doi:10.1371/journal.pone.0040946)
Supplement: Table S3 — A list of 14 cancer drugs that were not identified by our approach. (DOCX) [file pone.0040946.s003.docx]

| **Drug** | **Relation to target** | **Knowledge about target** | **Evidence** |
| --- | --- | --- | --- |
| capecitabine | TYMS inhibitor | Not captured |  |
| cladribine | PNP inducer | PNP positively regulates cell proliferation | PNP: positive regulation of T cell proliferation (GO:0042102) |
| cladribine | RRM2B inhibitor | RRM2B positively regulates apoptosis | RRM2B: induction of apoptosis (GO:0006917) |
| cladribine | POLE3 inhibitor | Not captured |  |
| cladribine | POLA1 inhibitor | Not captured |  |
| cladribine | POLE inhibitor | Not captured |  |
| cladribine | POLE2 inhibitor | Not captured |  |
| cladribine | POLE4 inhibitor | Not captured |  |
| cladribine | RRM1 inhibitor | RRM1 suppresses tumor activity | “ectopic expression of RRM1 suppresses proliferation of ras-transformed mouse fibroblasts, and high levels of RRM1 expression are associated with a significant survival benefit in patients with lung cancer” (PMID: 16818620) |
| cladribine | RRM2B inhibitor | RRM2B positively regulates apoptosis | induction of apoptosis (GO:0006917) |
| clofarabine | POLA1 inhibitor | Not captured |  |
| clofarabine | RRM1 inhibitor | RRM1 suppresses tumor activity | “ectopic expression of RRM1 suppresses proliferation of ras-transformed mouse fibroblasts, and high levels of RRM1 expression are associated with a significant survival benefit in patients with lung cancer” (PMID: 16818620) |
| Denileukin diftitox | IL2RB inducer | IL2RB negatively regulates apoptosis | IL2RB: positive regulation of survival gene product expression (GO:0045885) |
| Epirubicin | CDH1 inhibitor | Not captured |  |
| Floxuridine | TYMS inhibitor | Not captured |  |
| Gemcitabine | RRM1 inhibitor | RRM1 suppresses tumor activity | “ectopic expression of RRM1 suppresses proliferation of ras-transformed mouse fibroblasts, and high levels of RRM1 expression are associated with a significant survival benefit in patients with lung cancer” (PMID: 16818620) |
| Hydroxyurea | RRM1 inhibitor | RRM1 suppresses tumor activity | “ectopic expression of RRM1 suppresses proliferation of ras-transformed mouse fibroblasts, and high levels of RRM1 expression are associated with a significant survival benefit in patients with lung cancer” (PMID: 16818620) |
| Imiquimod | TLR7 inducer | Not captured |  |
| Leucovorin | TYMS inhibitor | Not captured |  |
| Levamisole | CHRNA3 inducer | Not captured |  |
| Pegfilgrastim | CSF3R inducer | Not captured |  |
| Vindesine | TUBB1 inhibitor | Not captured |  |
| Vinorelbine | TUBB inhibitor | TUBB positively regulars apoptosis | TUBB: natural killer cell mediated cytotoxicity (GO:0042267) |
